# Cerebrospinal-fluid Orexin-A levels in different neurocognitive disorders: a comparison study

**DOI:** 10.1007/s10072-025-08148-0

**Published:** 2025-04-08

**Authors:** Susana Lozano-Tovar, Riccardo Cremascoli, Marzia Nuccetelli, Giuseppe Sancesario, Stefania Cattaldo, Elisa Prina, Federico Verde, Simone Cappelli, Sergio Bernardini, Nicola Biagio Mercuri, Claudio Liguori

**Affiliations:** 1 Facultad de Psicología, Universidad Nacional Autónoma de México (UNAM), Circuito Ciudad Universitaria Avenida, C.U, Mexico City, 04510 Mexico; 2 Unit of Neurology and Neurorehabilitation, IRCCS, Istituto Auxologico Italiano, San Giuseppe Hospital, 28824 Verbania, Italy; 3 Department of Clinical Biochemistry and Molecular Biology, University of Rome Tor Vergata, Rome, Italy; 4 Department of Systems Medicine, University of Rome Tor Vergata, 00133 Rome, Italy; 5 Laboratory of Clinical Neurobiology, IRCCS, Istituto Auxologico Italiano, San Giuseppe Hospital, 28824 Verbania, Italy; 6 Department of Neurology and Laboratory of Neuroscience, IRCCS, Istituto Auxologico Italiano, Milan, Italy; 7 Department of Pathophysiology and Transplantation, “Dino Ferrari” Center, Università degli Studi di Milano, Milan, Italy; 8 Laboratory of Psychology, IRCCS, Istituto Auxologico Italiano, San Giuseppe Hospital, 28824 Verbania, Italy; 9 Sleep Medicine Centre, Neurology Unit, University Hospital of Rome Tor Vergata, Viale Oxford 81, 00133 Rome, Italy

**Keywords:** Orexin-A, β-amyloid, Alzheimer’s disease, FTD, iNPH, tau, Dementia, Cognitive impairment

## Abstract

In the present study, we investigated the differences in cerebrospinal fluid (CSF) orexin-A levels among patients with different neurocognitive disorders, such as mild or moderate to severe Alzheimer’s disease (AD; mAD, msAD, respectively), behavioral variants of frontotemporal dementia (bv-FTD), non-fluent primary aphasia (NFPA), and idiopathic normal pressure hydrocephalus (iNPH). A total of 214 participants were evaluated (mAD, 45; msAD, 31; bv-FTD, 12; NFPA, 22; iNPH, 13; non-demented elderly controls, 91). The highest CSF orexin-A levels were found in iNPH patients (263.31 ± 56.89 pg/mL). Patients affected by NFPA (210.86 ± 61.99 pg/mL), iNPH, and msAD (173.04 ± 19.76 pg/mL) showed higher CSF orexin-A concentrations than controls (145.18 ± 27.01pg/mL) (*p* < 0.001). Bv-FTD (190.12 ± 100.84 pg/mL) and mAD (130.76 ± 21.70 pg/mL) patients showed no significant differences in CSF orexin-A levels compared with controls. mAD patients showed also lower CSF orexin-A concentrations than all other patient groups.

In conclusion, orexin-A presents different CSF levels among neurocognitive disorders. The mechanisms underlying this difference vary and may include sleep-wake cycle impairment, behavioral disturbances, and CSF dynamics. The development of drugs that antagonize the orexin system could open a new frontier of research linking orexin neurotransmission to neurocognitive disorders.

**Supplementary Information:**

The online version contains supplementary material available at 10.1007/s10072-025-08148-0.

## Introduction

Orexin-A principally controls the sleep-wake cycle by maintaining wakefulness, although other physiological functions can be regulated by the circadian brain level fluctuations of this neurotransmitter [1]. Accordingly, the orexin-A system participates in the regulation of various physiological functions, including mood, stress, reward, eating, and cognition [1]. Orexin-A is released from the lateral hypothalamic neurons, which project to several brain regions [1]. The multifaceted functions of orexin-A are mediated by both widespread orexin projections and receptors distributed across the cortical, subcortical, and brainstem regions [1]. The orexin system has been principally reviewed in animal and human narcolepsy studies [2]. However, a role of orexin system in regulating cognitive performance has been documented [3]. Briefly, orexin system can interact with cholinergic and non-cholinergic neurons in particular in regulating the response to salient stimuli as well as the mechanisms underlying attention [4–5]. Moreover, the orexin system, as a potent endogenous arousal-promoting system, can impact on memory processes since orexin neurons project diffusely to the hippocampus [6]. As a consequence, orexin system activity and function have been evaluated in neurological disorders featured by cognitive impairment and dementia [7–8]. In the past decade, cerebrospinal fluid (CSF) orexin-A levels have been thus measured in patients with mild cognitive impairment (MCI) and dementia, particularly those with Alzheimer’s disease (AD) and frontotemporal dementia (FTD) [9–18]. Although in vivo studies have documented an increase in CSF orexin-A levels in patients with MCI and AD [9–11], undisputed evidence has not yet been achieved, and existing studies present conflicting results, since some of them are post-mortem studies and other reports included different other neurocognitive disorders not homogeneously classified according to the last published diagnostic criteria [12–14, 19]. Consistently, preliminary findings have failed to show any significant differences in CSF orexin-A levels between patients with FTD or idiopathic normal pressure hydrocephalus (iNPH) and controls [17, 20].

To better understand the clinical potential of the measurement of CSF orexin-A levels in patients with neurocognitive disorders and dementia, and considering the lack of studies in the literature comparing this biomarker in different dementias, in this retrospective study, we compared the orexin-A concentrations in CSF samples obtained from patients with AD, behavioral variant of FTD (bv-FTD), non-fluent primary aphasia (NFPA), and iNPH compared to a group of non-demented older controls.

## Materials and methods

### Participants

This multicenter study included patients retrospectively recruited from the Neurology Unit at the University Hospital of Rome Tor Vergata and the Units of Neurology and Neurorehabilitation at the Istituto Auxologico Italiano of Piancavallo between January 2012 and December 2015. Patients with AD, bv-FTD, NFPA, and iNPH were included after completing clinical and instrumental diagnostic work-up, leading to a diagnosis based on the diagnostic guidelines for each disorder. All diagnoses were reviewed during the manuscript preparation (2024). Patients with AD met the criteria for AD diagnosis according to recently published clinical criteria [21]. Patients with AD were further divided into two subgroups based on the Mini-Mental State Examination (MMSE) score: mild AD (mAD, MMSE ≥ 21) and moderate-severe AD (msAD, MMSE < 21) [22]. Patients were considered as affected by the behavioral variant of FTD (bv-FTD) according to the Neary et al. consensus criteria [23]. Moreover, a group of patients diagnosed as affected by the NFPA was also included in the study [24]. iNPH was diagnosed according to the iNPH guideline criteria, based on the results of clinical and brain magnetic resonance imaging (MRI), and confirmed by a positive spinal tap test [25–26].

All patients underwent a diagnostic workup, including history taking, physical and neurological examination, laboratory tests, standard neuropsychological evaluation, brain MRI, and lumbar puncture (LP) for CSF analysis. Eligible patients were also required to fulfil the following inclusion criteria: CSF collection performed in the morning, following a night of at least 6 h of sleep, and confirmation of the diagnosis at follow-up visits at each center. The exclusion criteria were as follows: intake of drugs active in the CNS at the time of LP; other neurological or psychiatric concomitant diseases; previous diagnosis of primary sleep disorders or other conditions interfering with sleep quality, such as chronic symptomatic obstructive pulmonary disease and epilepsy; history of brain trauma; and abnormal cell count (> 4 cells/mcL) in the CSF sample analysis.

The controls were inpatients in the same units who underwent LP for diagnostic purposes, and their clinical and instrumental data excluded CNS or systemic diseases. Moreover, the controls did not present objective cognitive impairment, brain atrophy on MRI, or sleep disorders and were drug-free at the time of CSF collection.

All procedures conformed to the tenets of the Declaration of Helsinki. The study protocol was approved by the ethics committees of the University Hospital of Rome Tor Vergata and the Istituto Auxologico Italiano of Piancavallo, and all participants enrolled in the study provided written informed consent.

### LP and CSF analysis

The patients and controls underwent LP between 8:00 and 10:00 am. Patients were punctured in the lateral position using atraumatic needles. The CSF was collected in polypropylene tubes using standard sterile techniques. Blood samples were obtained at the same time as the LP. Immediately following collection, CSF samples were stored on ice, sent to a local laboratory, and processed within 1 h. The first CSF sample (4 mL) was used for routine analysis, while the second (6 mL) and the third (6 mL) samples, were centrifuged to eliminate cells and cellular debris immediately after collection, and stored at -80 °C until the analysis to assess CSF levels of total-tau (t-tau), phosphorylated-tau (p-tau), β-amyloid_42_ (Aβ_42_) (all in the second sample), and orexin-A (in the third sample).

### CSF levels of Aβ_42_, t-tau, p-tau, and orexin-A measurement

The levels of t-tau, p-tau, Aβ_42_ were determined according to previously published standard procedures [27], using commercially available sandwich enzyme-linked immunosorbent assays (ELISA) kits (Innotest β-Amyloid 1–42, Innotest h-T-tau, InnotestPhospho-T-tau 181; Innogenetics, Ghent, Belgium). CSF levels of orexin-A were also detected using a commercially available ELISA kit (Orexin-A A/Hypocretin-1 EIA Kit; Phoenix Pharmaceuticals, Burlingame, CA, USA) [17].

### Data analysis

Statistical analyses involved the Mann–Whitney U test, the Kruskal-Wallis test, and the analysis of covariance (ANCOVA), performed as appropriate. In the ANCOVA, we used CSF biomarker levels as the dependent variable, with group as the fixed factor, and age and sex as covariates. Post-hoc analysis was performed using Dunn’s test with the Bonferroni correction. Correlations between all variables and CSF orexin-A levels were separately analyzed using the Spearman correlation test in SPSS 25.

## Results

### Demographic and clinical data of AD, NFPA, bv-FTD, iNPH and controls

In total, 214 participants were evaluated and divided into different groups. Based on the diagnostic criteria used, the following groups were included: 76 patients with AD, 34 with FTD (bv-FTD, 12; NFPA, 22), 13 with iNPH, and 91 non-demented older controls. AD patients were further distributed in two groups based on the cognitive status measured by the MMSE: mAD, including 45 subjects and msAD, including 31 subjects. The groups did not differ in terms of age, except for the comparison between the msAD and control group (Supplementary Table 1). Considering the MMSE data, all groups of patients showed lower MMSE score than controls. Considering the comparison between patient groups, patients with mAD showed higher MMSE score than those with msAD and bv-FTD (Supplementary Table 2). Demographic and clinical data are shown in Table 1.

**Table 1:** Sociodemographic, clinical and CSF data in all groups

		mAD	msAD	Bv-FTD	NFPA	iNPH	Controls
		*n* = 45	*n* = 31	*n* = 12	*n* = 22	*n* = 13	*n* = 91
Sociodemographic data*							
Sex	F	20	15	5	4	3	47
	M	25	16	7	18	10	44
Age Mean (SD)		69.71 (7.76)	72.42 (6.70)	66.92 (9.02)	69.14 (8.15)	74.0 (7.53)	61.33 (15.76)
MMSE Mean (SD)		23.24 (0.80)	17.90 (2.75)	17.75 (1.65)	20.73 (2.72)	20.54 (2.18)	29.21 (1.06)
CSF data (expressed in pg/ml)*							
t-tau Mean (SD)		608.87 (230.44)	763.00 (277.75)	320.917 (221.44)	543.45 (533.67)	168.31 (54.97)	226.01 (76.34)
p-tau Mean (SD)		90.98 (37.07)	105.06 (47.31)	48.58 (36.81)	59.82 (37.06)	28.15 (15.32)	43.99 (11.18)
Aβ_42_ Mean (SD)		330.73 (255.35)	334.51 (212.62)	572.5 (307.43)	697.55 (371.95)	554.62 (196.39)	943.68 (198.12)
Orexin-A Mean (SD)		130.76 (21.70)	173.04 (19.76)	190.12 (100.84)	210.86 (61.99)	263.31 (56.89)	145.18 (27.01)

*Abbreviations*: F: female; M: male; mAD: Mild AD. msAD: Moderate-severe AD. bv-FTD: fronto-temporal dementia - behavioral variant. NFPA: Non-fluent primary aphasia. iNPH: idiopathic normal pressure hydrocephalus. MMSE: Mini Mental State Examiantion. T-tau: total tau. P-tau: phosphorylated tau. Aβ_42_: β-amyloid_42_. SD: Standard deviation

* Statistical analysis is reported in Table 2, and in the Supplementary Tables 1, 2, 3, 4, 5

### CSF levels of orexin-A in the different groups

**Fig. 1:**
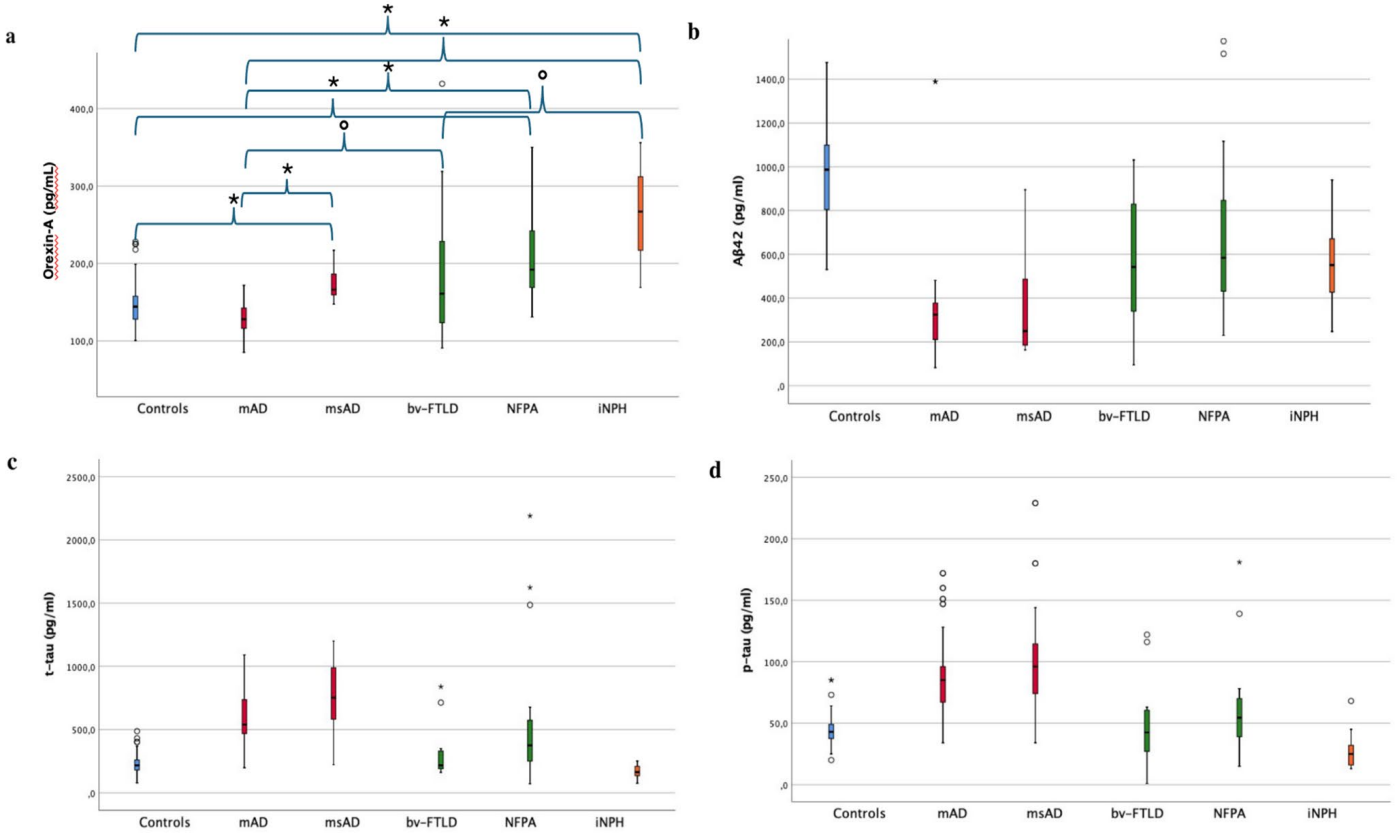
Comparison of CSF levels of orexin-A between groups. *^,^° indicates statistical significance of the comparison: ° *p* < 0.05; *p < 0.001. Results of the statistical analysis are all reported in Table 2

The iNPH group showed the highest CSF orexin-A levels (mean -ME- =263.31 pg/ml), while the mAD group showed the lowest concentration (ME = 130.76 pg/ml) (Table 1; Fig. 1a). Therefore, ANCOVA was performed to compare CSF orexin-A levels between all groups included in the study using age and sex as covariates. The first results revealed that CSF orexin-A levels were significantly different among all groups (*p* < 0.001). Therefore, a post-hoc analysis was performed using Dunn’s test with Bonferroni correction. Considering the comparison between controls and each neurocognitive disorder group, CSF orexin-A levels were significantly higher in patients with NFPA (*p* < 0.001), iNPH (*p* < 0.001), and msAD (*p* < 0.001) than in controls (Table 2). There were no significant differences between mAD or bv-FTD group and control group (Table 2).

**Table 2:** Comparisons in CSF orexin-A levels among controls and patients. ANCOVA analysis was performed and completed by using the post-hoc Dunn test corrected with the Bonferroni correction. Significance of the P value was set at *p* < 0.05

Comparison between Groups	*p*_Dunn_	*p*_bonf_
Controls - mAD	0.009	0.136
Controls - msAD	< 0.001	< 0.001
Controls– bv-FTD	0.061	0.912
Controls - NFPA	< 0.001	< 0.001
Controls - iNPH	< 0.001	< 0.001

*Abbreviations* mAD: mild AD. msAD: moderate-severe AD. bv-FTD: fronto-temporal dementia - behavioral variant. NFPA: Non-fluent primary aphasia. iNPH: idiopathic normal pressure hydrocephalus

Considering the comparison between groups of neurocognitive disorders, mAD patients presented lower CSF orexin-A levels than msAD (*p* < 0.001), bv-FTD (*p* = 0.04), NFPA (*p* < 0.001), or iNPH (*p* < 0.001) patients. Moreover, the iNPH group had higher CSF orexin-A levels than the bv-FTD group (*p* = 0.011). No other differences in CSF orexin-A levels were found between groups (Table 2; Fig. 1A).

The CSF levels of orexin-A were also compared between males and females in all groups, and statistically significant differences were found only in the control group in which males presented higher CSF orexin levels than females (Table 3).

**Table 3:** Comparisons in CSF orexin-A levels among neurocognitive disorder groups. ANCOVA analysis was performed and completed by using the post-hoc Dunn test corrected with the Bonferroni correction. Significance of the P value was set at *p* < 0.05

Comparison between Groups	*p*_Dunn_	*p*_bonf_
mAD - msAD	< 0.001	< 0.001
mAD– bv-FTD	0.003	0.040
mAD - NFPA	< 0.001	< 0.001
mAD - iNPH	< 0.001	< 0.001
msAD– bv-FTD	0.072	1.000
msAD - NFPA	0.109	1.000
msAD - iNPH	0.009	0.141
bv-FTD - NFPA	0.010	0.145
bv-FTD - iNPH	< 0.001	0.011
NFPA - iNPH	0.113	1.000

*Abbreviations* mAD: mild AD. msAD: moderate-severe AD. bv-FTD: fronto-temporal lobe dementia - behavioral variant. NFPA: Non-fluent primary aphasia. iNPH: idiopathic normal pressure hydrocephalus

**Table 4:** Comparison in CSF orexin-A levels between male and females of all groups

Groups	Sex				
	Female	Male	p value		
	Mean (SD)				
Controls	136.145 (22.89)	154.8 (28.34)	< 0.001		
mAD	133.28 (19.73)	128.744 (23.35)	0.51		
msAD	177.4 (20.99)	168.95 (18.24)	0.36		
bv-FTD	202.32 (90.00)	181.41 (114.15)	0.57		
NFPA	225.25 (73.38)	207.66 (61.13)	0.73		
iNPH	266.667 (77.53)	262.3 (54.54)	0.93		

*Abbreviations* mAD: mild AD. msAD: moderate-severe AD. bv-FTD: fronto-temporal dementia - behavioral variant. NFPA: Non-fluent primary aphasia. iNPH: idiopathic normal pressure hydrocephalus. SD: Standard deviation

* significance of Mann–Whitney U test set at *p* < 0.05

### CSF levels of Aβ_42,_ t-tau and p-tau in the different groups

CSF concentrations of Aβ_42_, t-tau and p-tau were measured in all groups (Table 1). The results of the statistical analysis are represented in Supplementary Tables 3, 4 and 5.

### Correlations between CSF levels of Aβ_42_ and orexin-A in patients and controls

We further studied the correlation between the CSF levels of Aβ_42_ and orexin-A in the control and patient groups, identifying a correlation between the CSF levels of Aβ_42_ and orexin-A only in controls (*r* = 0.26; *p* = 0.014). We did not find any correlation between CSF levels of Aβ_42_ and orexin-A in any of the patient groups.

### Correlations between CSF levels of t-tau and p-tau and orexin-A in patients and controls

CSF levels of orexin-A correlated with CSF t-tau concentrations in the control group (*r* = 0.37, *p* < 0.001). We did not find any correlation between CSF orexin-A levels and t-tau or p-tau levels in patients with mAD (*r*=-0.18; *p* = 0.24) or msAD (*r* = 0.40; *p* = 0.82). Similarly, no significant correlations were found between these biomarkers in any of the other groups (*p* > 0.05).

### Correlation between clinical data and CSF orexin-A levels in patient groups

By correlating the MMSE scores with the orexin-A CSF levels in the different groups, we found a significant negative correlation only in the mAD (*r*= -0.54; *p* < 0.001) and msAD (*r*= -0.92; *p* < 0.001) groups. No other correlations were found between these variables in the other groups.

## Discussion

This study investigated the CSF orexin-A levels in different neurocognitive disorders, in order to quantify the CSF levels of this neurotransmitter in patients affected by cognitive impairment and dementia. The main finding of the present study was the documentation of significant differences in CSF orexin-A levels among patients with msAD, NFPA, and iNPH compared to older controls. In particular, patients with iNPH presented with the highest mean CSF orexin-A concentrations, while patients with mAD showed the lowest mean CSF orexin-A levels. When comparing the groups of patients, subjects affected by iNPH showed higher CSF orexin-A levels than those with mAD and bv-FTD; conversely, patients with msAD showed higher CSF orexin-A levels than those with mAD; and patients with both NFPA and bv-FTD presented higher CSF orexin-A levels than patients with mAD.

Although previous studies showed higher CSF orexin-A concentrations in patients with neurocognitive disorders, most of them included only patients with AD compared to controls; therefore, a general agreement has not been yet achieved [9–18, 28–33]. Moreover, *post-mortem* studies performed in patients with AD documented a reduction in orexinergic neurons and significantly lower CSF orexin-A levels compared to controls [19, 34], suggesting that the in vivo documentation of high CSF orexin-A levels may reflect the modification of orexinergic neurotransmission depending on factors such as sleep-wake cycle dysregulation, feeding, and behavior more than the integrity of the orexin system [3, 35–37]. Accordingly, previous studies performed in patients with AD have documented that an increase in CSF orexin-A levels is correlated with the degree of cognitive impairment and the concomitant presence of neuropsychiatric symptoms, sleep impairment, and sleep-wake cycle misalignment [9–10, 38, 39, 40]. This interplay between CSF orexin-A concentrations and the wide symptomatology presented by patients affected by AD principally considers the effect of the increase in nighttime wakefulness, reduction of REM sleep, and loss of the sleep-wake cycle, which has been well-documented in AD [3–4]. Accordingly, orexin-A could be considered a neurotransmitter with dynamic secretion in relation to different stimuli; therefore, the increase in wakefulness, reduction in sleep (particularly REM sleep), and loss of circadian regulation of the sleep-wake cycle are all associated with an increase in CSF orexin-A levels [9–10]. In the present study, we confirmed an increase in CSF orexin-A concentration in msAD compared to mAD, and a correlation between CSF orexin-A levels and MMSE scores in patients with mAD or msAD.

Considering previous studies performed in patients with FTD, a correlation between CSF orexin-A concentration and disease-related clinical symptoms has been documented. Consistently, higher levels of excessive daytime sleepiness (EDS), subjectively investigated using the Epworth Sleepiness Scale, were correlated with a decrease in CSF orexin-A levels in a small group of subjects with FTD not further characterized according to the symptoms presented by patients [17]. Conversely, in a study involving patients with bv-FTD, symptoms of impulsivity and the presence of extrapyramidal signs were found to be associated with high CSF orexin-A levels [33]. These findings, not yet further confirmed by following studies, showed that EDS was associated with a reduction in CSF orexin-A levels, whereas impulsivity and extrapyramidal signs were associated with an increase in CSF orexin-A concentrations, which could explain the very high spread of CSF orexin-A levels documented in the patients with bv-FTD and NFPA included in this study (see Fig. 1). Notably, numerous orexinergic projections originating from the hypothalamic area are present in the frontal lobe [3, 35–37].

Patients with iNPH had the highest mean CSF orexin-A levels, which were higher than those of controls. A single study investigated CSF orexin-A levels in three patients with iNPH, and documented that two of them presented with intermediate levels (123 and 168 pg/ml, respectively), and one with very high levels (1142 pg/ml) [20]. Considering the results of the present study, it is challenging to explain the high CSF levels of orexin-A in patients with iNPH. A previous study documented a high prevalence of sleep-disordered breathing (SBD) in patients with iNPH [41], while another showed that CSF orexin-A levels could be higher in patients with SDB than in controls [31]. Another main trigger for high CSF orexin-A levels could be the deficient glymphatic system function documented in patients with iNPH. In agreement with this evidence, one study based on the analysis of brain MRI of patients with iNPH documented reduced perivascular influx and efflux of intrathecally injected contrast agents compared to controls, suggesting that the impairment of glymphatic function in iNPH may also be responsible for the increased CSF levels of peptides (including orexin-A) [42]. Moreover, the rostro-caudal dynamics of orexin-A in the CSF [the relationship between ventricular (rostral) and lumbar (caudal) levels] have already been hypothesized in patients with iNPH, who showed significantly higher lumbar than ventricular orexin-A levels [43]. As such, a combination of different factors (high prevalence of SDB, glymphatic system malfunction, and rostro-caudal dynamics of CSF orexin-A levels) can contribute to the significant increase in CSF orexin-A levels in iNPH.

Overall, the present study illustrates the CSF orexin-A levels in different neurocognitive disorders, including cognitive impairment, neuropsychiatric symptoms, and dementia. Patients were recruited from two centers, thereby increasing the significance of the results. Orexin-A is a neurotransmitter that mainly regulates the sleep-wake cycle; however, in addition to its regulatory role in sleep and wakefulness, its role in the regulation of attention, cognition, and behavior has also been widely recognized [3, 35–37]. As such, measurement of CSF orexin-A concentrations could be considered in the diagnostic workup of patients with cognitive impairment and dementia, considering the possibility of correlating CSF levels of this neuropeptide with neuropsychiatric symptoms, with particular attention to sleep and the sleep-wake cycle. Further, dual orexin-A receptor antagonists (DORAs) have recently been approved for the treatment of chronic insomnia disorder, and their role in counteracting the neurodegenerative processes by antagonizing the orexinergic system has been already hypothesized in AD patients [44]. Therefore, further investigations on the role of orexin-A on symptoms presented by patients with cognitive impairment and dementia should be planned also for understating the clinical potential of including DORAs in the therapeutic armamentarium available for patients with neurocognitive disorders.

This study has several limitations that should be considered. First, due to the retrospective nature of the study, standardized cognitive and behavioral evaluations were not collected and thus not correlated with CSF orexin-A levels. Second, older controls included individuals with subjective cognitive symptoms without any other signs or symptoms of neurodegenerative disorders; as such, further validation using age-matched healthy controls should be planned in the future. Third, the groups of subjects included in the study were not matched for age and sex. Although the analyses included covariates for these parameters, further studies should be planned to confirm these preliminary findings, possibly including larger groups of patients from multiple sites, to increase the significance of the results presented here. Finally, the patients included in the present study were divided in several subgroups, potentially reducing its statistical power.

In conclusion, considering the importance of identifying biomarkers to discriminate between different neurological disorders characterized by cognitive impairment, the present study proposes the clinical potential of adding CSF orexin-A levels to the list of CNS biomarkers already measured in patients with neurocognitive disorders, in the view of developing a precision medicine-based approach.

## Electronic supplementary material

Below is the link to the electronic supplementary material.

Supplementary Material 1
