# Supplementary material for: Cerebrospinal-fluid Orexin-A levels in different neurocognitive disorders: a comparison study
Source: Neurol Sci. 2025 Apr 8;46(8):3631–8. doi: 10.1007/s10072-025-08148-0 (PMC12267319; doi:10.1007/s10072-025-08148-0)
Supplement: Supplementary file 1 — Supplementary Material 1 [file 10072_2025_8148_MOESM1_ESM.docx]

**Supplementary Tables**

Supplementary tables: (1) Age comparison between groups. (2) MMSE comparison between groups. (3-5) Comparison of CSF levels of Aβ_42,_ (2), t-tau (3), p-tau (4) between groups.

All the analysis were performed using the ANCOVA and post-hoc analyses were performed using the Dunn test corrected by the Bonferroni correction.

Supplementary Table 1. Age comparison between groups.

| **Dunn's Post Hoc Comparisons** | | |
| --- | --- | --- |
| **Comparison** | ***p*** | ***p _bonf_*** |
| Controls - mAD | 0.005 | 0.075 |
| Controls - msAD | < .001 | 0.003 |
| Controls – bv-FTD | 0.292 | 1.000 |
| Controls - NFPA | 0.069 | 1.000 |
| Controls - iNPH | 0.004 | 0.060 |
| mAD - msAD | 0.131 | 1.000 |
| mAD – bv-FTD | 0.177 | 1.000 |
| mAD - NFPA | 0.339 | 1.000 |
| mAD - iNPH | 0.157 | 1.000 |
| msAD – bv-FTD | 0.049 | 0.736 |
| msAD - NFPA | 0.095 | 1.000 |
| msAD - iNPH | 0.433 | 1.000 |
| bv-FTD - NFPA | 0.299 | 1.000 |
| bv-FTD - iNPH | 0.061 | 0.920 |
| NFPA - iNPH | 0.113 | 1.000 |

***Abbreviations:*** *mAD: mild AD. msAD: moderate-severe AD. bv-FTD: fronto-temporal dementia - behavioral variant. NFPA: Non-fluent primary aphasia. iNPH: idiopathic normal pressure hydrocephalus.*

Supplementary Table 2. MMSE comparison between groups.

| **Dunn's Post Hoc Comparisons** | | |
| --- | --- | --- |
| **MMSE Comparison** | **p** | **p _bonf_** |
| Controls - mAD | < .001 | < .001 |
| Controls - msAD | < .001 | < .001 |
| Controls – bv-FTD | < .001 | < .001 |
| Controls - NFPA | < .001 | < .001 |
| Controls - iNPH | < .001 | < .001 |
| mAD - msAD | < .001 | < .001 |
| mAD – bv-FTD | < .001 | 0.006 |
| mAD - NFPA | 0.023 | 0.349 |
| mAD - iNPH | 0.038 | 0.574 |
| msAD – bv-FTD | 0.393 | 1.000 |
| msAD - NFPA | 0.044 | 0.656 |
| msAD - iNPH | 0.093 | 1.000 |
| bv-FTD - NFPA | 0.057 | 0.848 |
| bv-FTD - iNPH | 0.093 | 1.000 |
| NFPA - iNPH | 0.455 | 1.000 |

***Abbreviations:*** *mAD: mild AD. msAD: moderate-severe AD. bv-FTD: fronto-temporal dementia - behavioral variant. NFPA: Non-fluent primary aphasia. iNPH: idiopathic normal pressure hydrocephalus.*

Supplementary Table 3. CSF Aβ_42_ levels comparison between groups.

| **Dunn's Post Hoc Comparisons** | | |
| --- | --- | --- |
| **Comparison** | **p** | **p _bonf_** |
| Controls - mAD | < .001 | < .001 |
| Controls - msAD | < .001 | < .001 |
| Controls - bv-FTD | < .001 | 0.006 |
| Controls - NFPA | 0.003 | 0.049 |
| Controls - iNPH | < .001 | 0.003 |
| mAD - msAD | 0.479 | 1.000 |
| mAD - bv-FTLD | 0.015 | 0.220 |
| mAD - NFPA | < .001 | < .001 |
| mAD - iNPH | 0.015 | 0.219 |
| msAD - bv-FTD | 0.017 | 0.256 |
| msAD - NFPA | < .001 | < .001 |
| msAD - iNPH | 0.017 | 0.258 |
| bv-FTD - NFPA | 0.154 | 1.000 |
| bv-FTD - iNPH | 0.479 | 1.000 |
| NFPA - iNPH | 0.135 | 1.000 |

***Abbreviations:*** *mAD: mild AD. msAD: moderate-severe AD. bv-FTD: fronto-temporal dementia - behavioral variant. NFPA: Non-fluent primary aphasia. iNPH: idiopathic normal pressure hydrocephalus.*

Supplementary Table 4. CSF t-tau levels comparison between groups.

| **Dunn's Post Hoc Comparisons** | | |
| --- | --- | --- |
| **Comparison** | **p** | **p _bonf_** |
| Controls - mAD | < .001 | < .001 |
| Controls - msAD | < .001 | < .001 |
| Controls - bv-FTD | 0.136 | 1.000 |
| Controls - NFPA | < .001 | 0.002 |
| Controls - iNPH | 0.057 | 0.852 |
| mAD - msAD | 0.143 | 1.000 |
| mAD - bv-FTD | < .001 | 0.003 |
| mAD - NFPA | 0.012 | 0.173 |
| mAD - iNPH | < .001 | < .001 |
| msAD - bv-FTD | < .001 | < .001 |
| msAD - NFPA | 0.001 | 0.020 |
| msAD - iNPH | < .001 | < .001 |
| bv-FTD - NFPA | 0.062 | 0.931 |
| bv-FTD - iNPH | 0.022 | 0.329 |
| NFPA - iNPH | < .001 | < .001 |

* p<.05, ** p< .01, *** < .001

***Abbreviations:*** *mAD: mild AD. msAD: moderate-severe AD. bv-FTD: fronto-temporal dementia - behavioral variant. NFPA: Non-fluent primary aphasia. iNPH: idiopathic normal pressure hydrocephalus.*

Supplementary Table 5. CSF p-tau levels comparison between groups.

| **Dunn's Post Hoc Comparisons** | | |
| --- | --- | --- |
| **Comparison** | **p** | **p _bonf_** |
| Controls - mAD | < .001*** | < .001*** |
| Controls - msAD | < .001*** | < .001*** |
| Controls - bv-FTD | 0.417 | 1.000 |
| Controls - NFPA | 0.031 | 0.470 |
| Controls - iNPH | 0.006** | 0.094 |
| mAD - msAD | 0.208 | 1.000 |
| mAD - bv-FTD | < .001*** | < .001*** |
| mAD - NFPA | < .001*** | 0.009** |
| mAD - iNPH | < .001*** | < .001*** |
| msAD - bv-FTD | < .001*** | < .001*** |
| msAD - NFPA | < .001*** | 0.002** |
| msAD - iNPH | < .001*** | < .001*** |
| bv-FTD - NFPA | 0.143 | 1.000 |
| bv-FTD - iNPH | 0.022* | 0.332 |
| NFPA - iNPH | < .001*** | 0.006** |

* p<.05, ** p< .01, *** < .001

***Abbreviations:*** *mAD: mild AD. msAD: moderate-severe AD. bv-FTD: fronto-temporal dementia - behavioral variant. NFPA: Non-fluent primary aphasia. iNPH: idiopathic normal pressure hydrocephalus.*
